# Quality of life, symptoms and dietary habits in oncology outpatients with malnutrition: A cross-sectional study

**DOI:** 10.1007/s12032-021-01460-7

**Published:** 2021-02-04

**Authors:** Mira Sonneborn-Papakostopoulos, Clara Dubois, Viktoria Mathies, Mara Heß, Nicole Erickson, Thomas Ernst, Jutta Huebner

**Affiliations:** 1 Klinik für Innere Medizin II, University Hospital Jena, Jena, Germany; 2 Comprehensive Cancer Center CCCLMU, Ludwig-Maximilian University Clinic, Munich, Germany; 3 UniversitätsTumorCentrum Jena, University Hospital Jena, Bachstraße 18, 07743 Jena, Germany

**Keywords:** Malnutrition, Cancer, Dietary habits, Screening, Side effects of cancer treatments

## Abstract

Cancer-related malnutrition has a high prevalence, reduces survival and increases side effects. The aim of this study was to assess oncology outpatients and risk of malnutrition. Reported symptoms and quality of life (QoL) in patients found to be at risk of malnutrition or malnourished were compared to patients without malnutrition. Using a standardized questionnaire, the European Organization for Research and Treatment of Cancer Questionnaire for Quality of Life and the Mini Nutritional Assessment (MNA), patients in an outpatient cancer clinic undergoing chemotherapy treatment at a German University Hospital were assessed for nutrition, risk of malnutrition and quality of life. Based on the MNA, 39 (45.9%) patients were categorized as malnourished or at risk for malnutrition. Loss of appetite (*n* = 37.6%, *p* < 0.001) and altered taste sensation (*n* = 30,3%, *p* < 0.001) were the symptoms most frequently associated with reduced food intake. Patients with risk of malnutrition scored lower on the global health status (*n* = 48.15%, *p* = 0.001). Side effects of cancer treatments lead to a higher risk of malnutrition and as a consequence lower QoL. These side effects should be addressed more efficiently in cancer care.

## Introduction

Many cancer patients suffer from malnutrition: a lack of adequate calories, protein, or other nutrients needed for tissue maintenance and repair [1]. This condition is defined by The European Society of Clinical Nutrition and Metabolism (ESPEN) by two options. Body mass index (BMI, kg/m^2^) <18.5 or the combination of unintentional weight loss (either >10% of habitual weight indefinite of time, or > 5% over 3 months) combined with either a reduced age specific BMI or a low fat free mass index using sex specific cut-offs [2]. For cancer patients, malnutrition is considered to be an important prognostic factor. Affected patients suffer from a negative clinical outcome up to premature death [3–9]. About 20–30% of patients with malignancies die due to tumor-related malnutrition [10]. Reduced quality of life is another important aspect associated to a poor nutritional status [11–13]. Therefore, malnutrition is highly relevant for everyday clinical practice in oncology and effective assessment, prevention, intervention and reevaluation are of major importance.

Despite the awareness that nutrition-related sides effects contribute largely to a reduced energy intake during cancer therapies, physicians often oversee nutritional issues, have limited knowledge how to calculate nutrition needs and do not routinely refer patients to a dietitian [14–18]. A variety of validated tools are a reliable method to detect the risk of malnutrition in cancer patients [19–21]. These include the Patient Generated-Subjective Global Assessment (PG-SGA), the Malnutrition Universal Screening Tool (MUST), the Nutritional Risk Screening (NRS-2002) and the Mini Nutritional Assessment (MNA). All these tools rely on measurable items like weight loss over defined time periods and assess the presence of symptoms associated with the disease [21, 22]. All of these tools are non-invasive, quick to apply, low in cost, easily feasible, and can be performed by any health professional. The PG-SGA short form is the only one which is completed by the patient, thus appropriate in settings where health professional’s time is limited. Du et al. compared screening tools and proposed that not only a comprehensive nutritional assessment tool should be recommended to all cancer patients but also that none of them should be implemented without follow-up care [23].

In a cross-sectional investigation, we aimed to compare differences in patient-reported symptoms regarding nutritional issues in patients with cancer undergoing chemotherapy who were well nourished compared to the patients identified at-risk of malnutrition or malnourished according to the MNA criteria [5, 24]. We also explored quality of life for both groups in order to evaluate the association of malnutrition and wellbeing. The results should help increase oncologists’ awareness to the importance of systematic screening for malnutrition, repeated re-evaluation during treatment, and to symptoms associated with malnutrition. Furthermore, the data may provide insights which strategies should be prioritized in the prevention of malnutrition through early interventions.

## Patients and methods

### Patients

During a two-week period in February 2018, 110 patients with any cancer undergoing chemotherapy treatment were asked to complete the questionnaires during their visit to the outpatient clinic of oncology at the University Hospital in Jena.

All patients attending chemotherapy during these days were asked to participate. Inclusion criteria were limited to patients ≥18 years undergoing chemotherapy for malignancy with the ability to understand the questions, and the willingness to participate. All MNA were performed by the same scientist. Afterwards the patient was requested to complete the questionnaires while waiting for the appointment and was visited again by the researcher to collect the form subsequently. Patients were informed about the anonymity of the data and data protection laws were respected. The study was approved by the ethics commission of the University Hospital Jena (Number of the ethical vote: 2019–1585).

### Outcome measures

#### Demographic and dietary information

Participant demographic data, including age, sex, height, current weight and weight before diagnosis, highest education, living situation was collected using a researcher-developed questionnaire filled in by the patient. Furthermore, the form included researcher-developed questions regarding the nutritional situation containing general information about diet habits in order to get an indication about the following: changes in nutrient intake before and after diagnosis, frequency of eating less following diagnosis to determine energy intake, information previously received about nutrition. In addition, it was asked if a nutrition counseling was performed to identify quality and source of knowledge and desire to involve in the nutritional situation as indication for compliance. Symptoms regarding nutritional intake to discover potentially influencing factors leading to reduced food intake have been obtained.

#### Quality of life

The European Organization for Research and Treatment of Cancer (EORTC QLQ-C30 version 3.0) questionnaire, which was developed specially for cancer patients and comprises 30 questions regarding quality of life, was also included. The 30 questions contained multi-item scales and single-item measures. It is composed of a global health status, five functional scales, three symptom scales and six symptom single items. Scores range from 0 to 100. For the global health status, a high score represents a high quality of life and for the functional scales a high score represents a high/ healthy level of functioning. For the symptom scales and items high scores represent a high level of symptoms/ problems [25].

#### Nutritional status

The long version of the Mini Nutritional Assessment in German was chosen as the third questionnaire. This questionnaire begins with six questions and produces a numerical score. Depending on the answer to these six questions, a further assessment consisting of 10 questions should be completed. These questions are completed if the total score of the answers to the first six questions add up to 11 points or more. The second section has a total of 16 points. Mid-arm circumference and calf circumference needs to be measured which was performed at the dominant arm on the same side of the body. A score of >23.5 indicates a well-nourished patient. A person is categorized as at risk for malnutrition when 17 to 23.5 points are reached and considered malnourished when less than 17 points are reached in the total assessment.

### Statistical analysis

Data from the questionnaires were transferred into IBM SPSS Statistics 25. Results were computed as means and standard deviation for quantitative variables and frequencies and percentages for qualitative variables. Correlations were tested to compare quantitative variables by Chi-square and t-test (*p*-value < 0.05). Chi-square test was used for comparison of data in nominal scale to investigate a possible association between frequency of eating less/ change in dietary habits/ avoiding products/ receiving information/ taking vitamin supplements and existing symptoms in malnourished and not malnourished patients. Results of the EORTC QLQ-C30 were generated into scores within the range of 0 to 100. According to EORTC guidelines overall scores were calculated and compared to gender and MNA categories using t-test [25].

## Results

### Demographic data

One hundred and nine patients were interviewed and completed the questionnaire (70 females (64.2%), 39 (35.8%) males). The median age was 61 years (SD = 12.4 years) (see Table 1). Breast cancer and lymphoma/leukemia were the most common diagnoses with 25 patients (22.9%) each, followed by 22 gastrointestinal (20.2%) and 15 urogenital (13.8%) cancers. Ninety-one patients (83.5%) lived with their family or partner and only 11 lived alone (10.1%), three were in a nursing home (2.8%). Further demographic data are shown in Table 1.

**Table 1:** Characteristics of study group (*N* = 109)

	n (%)
*Gender*	109 (100)
Female	70 (64.2)
Male	39 (35.8)
*Age*	107 (98.2)
Minimum	32
Maximum	87
Median	61
≤ 40 years	9 (8.3)
41–50 years	11 (10.1)
51–60 years	30 (27.5)
61–70 years	32 (29.4)
71–80 years	20 (18.3)
≥ 81 years	5 (4.6)
*Diagnosis (type of cancer)*	106 (97.2)
Breast	25 (22.9)
Lymphomas/Leukemia	25 (22.9)
Gastrointestinal	22 (20.2)
Urological/Urogenital	15 (13.8)
Gynecological	7 (6.4)
Lung	6 (5.5)
Head and Neck	2 (1.8)
Other	4 (3.7)
*Years from diagnosis*	103 (94.5)
> 10 years ago	4 (3.7)
2 to 10 years ago (2007–2016)	22 (20.3)
Last year (2017)	44 (40.4)
Current year (2018)	5 (4.6)
*Living situation*	105 (96.3)
Alone	11 (10.1)
With partner	72 (66.1)
With family	19 (17.4)
Nursing home	3 (2.8)
*Level of education*	107 (98.2)
Primary school	14 (12.8)
Middle school leaving certificate (9th or 10th grade)	17 (15.6)
High school diploma	3 (2.8)
Vocational training	33 (30.3)
University degree	40 (36.7)
*Physical activity*	104 (95.4)
No	64 (58.7)
1-2x at least 30 min per week	23 (21.1)
3-4x at least 30 min per week	10 (9.2)
> 4x at least 30 min per week	7 (6.4)
*Do you smoke?*	105 (96.3)
Yes	12 (11)
No	93 (85.3)
*How often do you drink alcohol?*	103 (94.5)
Never	46 (42.2)
1x per month or less	25 (22.9)
2-3x per month	14 (12.8)
1-2x per week	12 (11)
3-4x per week	3 (2.8)
Every day	3 (2.8)

Seventy-nine patients (72.4%) reported having had a change of weight after diagnosis. Sixty-five patients (59.6%) lost weight (mean 10 kg) while 14 reported having gained weight (12.8%; mean 6.6 kg) (Table 2). There were no significant correlations in terms of demographic data (gender, age, cancer entity, year of diagnosis or level of education). Significantly more patients with weight loss reported having changed their nutrition habits after diagnosis (*p* = 0.039).

**Table 2:** Body weight changes and amount of loss/gain in kilograms (*N* = 96)

	n (%)
*Weight change*	96 (88.1)
No weight change	17 (15.6)
Weight loss	65 (59.6)
Weight gain	14 (12.8)
*Weight loss after diagnosis*	65 (59.6)
Mean [IQR]	10 [4–14]
≤ 5 kg	27 (24.8)
6-10 kg	16 (14.7)
11-15 kg	8 (7.3)
16-20 kg	6 (5.5)
21-25 kg	4 (3.7)
> 25 kg	4 (3.7)
*Weight gain after diagnosis*	14 (12.8)
Mean [IQR]	6.6 [3–8.5]
≥ 5 kg	7 (6.4)
6-10 kg	5 (4.6)
> 11 kg	2 (1.8)

BMI varied slightly among the distribution of the different BMI classes in the whole collective. In total 41 patients had a change in weight after diagnosis resulting in a shift of their BMI. Whereas 35 lost weight resulting in a lower BMI and only 6 had a gain of weight resulting in higher BMI, respectively.

The MNA screening and consecutive full MNA were completed by 85 patients (78.0%). In total, the assessment revealed 34 patients at risk of malnutrition (31.2%) and 5 were considered to be malnourished (4.6%). 46 patients (42.2%) had a normal nutritional status.

### Reduced dietary intake

One hundred and three patients (94.5%) completed the data relevant to dietary intake. Twenty-seven patients (24.8%) reported eating less than before diagnosis at least once a week and 18 (16.5%) reported doing so every day.

**Fig. 1:**
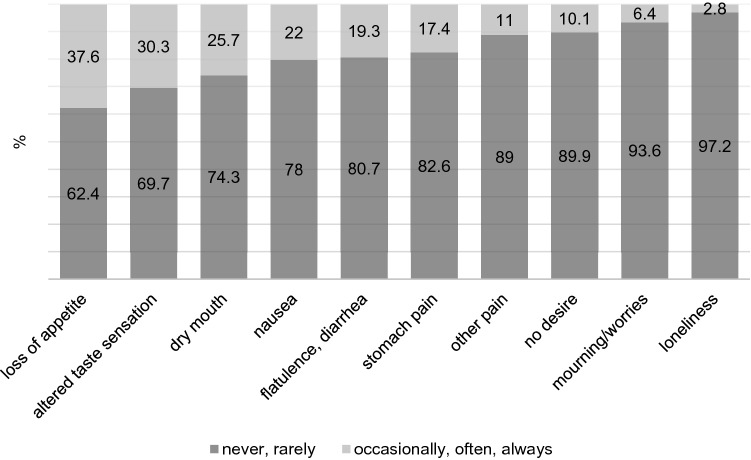
Participants who reported frequency of symptoms as reasons for eating less (*N* = 109)

Figure 1 shows the frequency of symptoms reported as reasons for eating less. The most frequent problems were loss of appetite (41 (37.6%)) followed by altered taste sensation (33 (30.3%)) and dry mouth (28 (25.7%)). Several symptoms correlated with eating less: nausea (*p* = 0.006, φ = 0.399), loss of appetite (*p* < 0.001, φ = 0.586), altered taste sensation (*p* < 0.001, φ = 0.469), stomach pain (*p* = 0.036, φ = 0.340), other pain (*p* = 0.004, φ = 0.408), no desire (*p* = 0.007, φ = 0.394), mourning/worries (*p* = 0.004, φ = 0.410) and flatulence/diarrhea (*p* = 0.013, φ = 0.375) (Fig. 1).

### Dietary habits

Seventy-five patients (68.8%) reported that they did not adhere to a special diet whereas 30 patients (27.5%) reported special dietary patterns such as vegetarian, no dairy, low carbohydrate, or no sugar. Among these patients, 27 reported not having followed any special diets before diagnosis (24.8%; *p* < 0.001, φ = 0.372). A change in dietary habits was associated with several side effects of cancer therapy. Considerably more patients with loss of appetite (*p* = 0.028, φ = 0.220) and altered taste sensation (*p* = 0.035, φ = 0.211) reported having changed dietary habits. Significantly more patients with altered taste sensation reported adhering to a special diet (*p* = 0.006, φ = 0.268). Patients who reported having received information on nutrition significantly more often wanted to be more involved with their nutrition decisions in the near future (*p* = 0.049, φ = 0.283). Thirty-one patients (28.4%) additionally stated that there were some foods which they avoided since diagnosis. Avoiding some products was correlated to the following symptoms: altered taste sensations (*p* = 0.005, φ = 0.275), stomach pain (*p* = 0.004, φ = 0.197), no desire to eat (*p* = 0.001, φ = 0.320) and flatulence/diarrhea (*p* = 0.019 each, φ = 0.231). Thirty-two patients (29.4%) reported taking vitamin supplements. Significantly more patients with the symptoms of loss of appetite (*p* = 0.004, φ = 0.197), dry mouth (*p* = 0.016, φ = 0.238), stomach pain (*p* = 0.013, φ = 0.244), no desire (*p* = 0.014, φ = 0.243) and flatulence/diarrhea (*p* = 0.042, φ = 0.201) took vitamin supplements.

### EORTC QLQ-C30

The EORTC QLQ-C30 global health status showed a mean score of 52.72 with no significant differences between male and female participants. Patients with risk of malnutrition or manifest malnutrition in the MNA showed lower global health status than those without risk (mean value 43.8 vs. 58.93; *p* = 0.001).

Mean values of all five functional scales are shown in Table 3. There were no significant differences between male and female participants. The MNA revealed patients with risk of malnutrition and malnourished patients had significantly lower scores for all functional scales (*p* values shown in Table 3). Symptom scales are shown in Table 3. Fatigue (51.89) scored highest and nausea and vomiting scored lowest of all symptom items (11.9). Significant differences between male and female participants were only seen with regard to financial difficulties (27.03 vs. 15.00; *p* = 0.048). Patients with risk of malnutrition scored significantly higher for all symptoms except dyspnea and diarrhea comparing the MNA groups. Additionally, financial difficulties and nausea/vomiting were not significantly correlated with nutrition risk (*p*-values shown in Table 3).

**Table 3:** Differences in EORTC QLQ-C30 scores of global health status, functional scales, symptom scales and measures between patients with and without malnutrition regarding the MNA (*N* = 91)

	Mean independent of nutritional status	MNA: Risk of malnutrition/ malnourished	MNA: Normal nutritional status	*p*-value
Global health status	52.7	43.80	58.93	0.001
Role functioning	51.7	40.95	63.95	0.003
Social functioning	57.1	47.75	70.54	0.002
Physical functioning	67.2	58.92	77.73	<0.001
Emotional functioning	68.7	61.62	77.50	0.004
Cognitive Functioning	78.2	70.72	87.70	0.001
Fatigue	51.9	61.59	41.80	0.002
Appetite loss	35	53.51	18.18	<0.001
Insomnia	34.2	45.61	27.41	0.01
Pain	34.7	44.74	18.22	<0.001
Dyspnoea	31.7	35.14	24.24	0.115
Financial difficulties	19.6	24.56	15.50	0.159
Constipation	17.6	21.93	6.35	0.007
Diarrhea	15.1	16.22	12.40	0.496
Nausea and vomiting	11.9	14.91	7.20	0.058

### Information

**Fig. 2:**
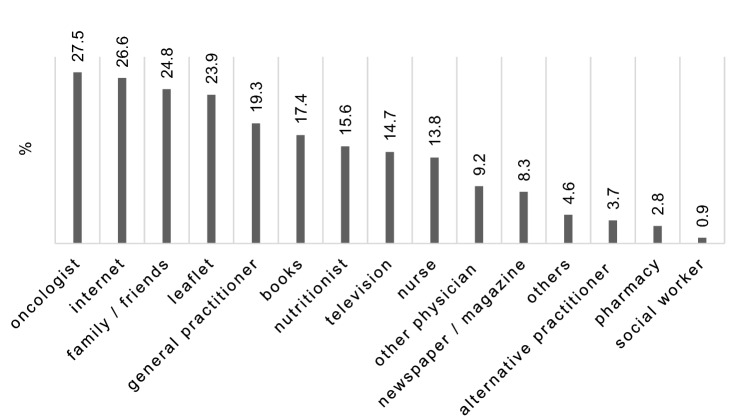
Source of information about nutrition regarding the type of cancer (*N* = 109)

In total, only 19 patients (17.4%) reported having received nutrition counseling. Significantly more men (12 (11.5%)) than women (7 (6.7%)) received counseling (*p* = 0.04, φ = 0.284). However, there were no statistically significant differences regarding weight loss, BMI or MNA category between patients who received and patients who did not receive nutrition counseling. Seventy-eight patients (71.6%) received nutrition information about nutrition and cancer. Twenty-four patients (22%) reported not to have received any information at all. Mostly the oncologist provided this information (30 (27.5%)). Only 17 patients (15.6%) got information from a nutritionist. Forty-one patients (37.6%) intended to focus on nutrition in the near future and 29 took this into consideration (26.6%). Significantly more patients with nutrition associated impact symptoms such loss of appetite (*p* = 0.002, φ = 0.38), altered taste sensation (*p* = 0.034, φ = 0.29), dry mouth (*p* = 0.046, φ = 0.279) and flatulence/diarrhea (*p* = 0.004, φ = 0.36) reported interest in nutrition. Patients who received no information on nutrition were significantly more often at risk of malnutrition (*p* = 0.008, φ = 0.292) (see Fig. 2).

### Mini nutritional assessment

There were no significant correlations between the groups of the MNA classification and the demographic data. Patients in the MNA risk group significantly reported more weight loss (*p* = 0.003, φ = 0.383). Significantly more patients with risk of malnutrition also reported having changed their nutritional habits after diagnosis (18 (47.4%) vs. 8 (18.2%); *p* = 0.005, φ = 0.313). In the group with risk of malnutrition significantly more patients took vitamin supplements as in the group with no risk (16 (42.1%) vs. 9 (20%) (*p* = 0.029), φ = 0.240).

## Discussion

The aim of this study was to assess the prevalence of malnutrition in cancer patients in an outpatient setting and to analyze factors associated with malnutrition and impact on quality of life. Our data show that there is still a high number of patients dealing with malnutrition and that malnutrition could be connected to reduced food intake due to nutrition associated impact symptoms. Additionally, we could confirm a high correlation to a poorer quality of life.

Overall, prevalence of suspected and manifested malnutrition was 45.9% among our patients. These results are higher than those reported by Calderon et al. [26] who revealed 36.4% of affected patients undergoing cancer surgery and initiated adjuvant chemotherapy using the Malnutrition Screening Tool (MST). Considering the prevalence of malnutrition at time of cancer diagnosis in outpatients settings, Alvaro Sanz et al. [27] reported 21.4% of patients being affected who were diagnosed with solid tumors from early diagnosis to advanced stages with begin of chemotherapy using Nutriscore to identify nutritional risk. Whereas another larger study using the MNA to assess malnutrition recorded prevalence of 43% in patients at first medical oncology visit [28]. However, the large range must be interpreted carefully. One reason for these differences are the miscellaneous populations and various tools used to determine the nutritional condition of cancer patients [22, 29].

In contrast to other recent studies we could not show a higher risk of malnutrition for older patients. Still our results appear to be similar to the data of Zhang et al. who assessed the MNA for older patients undergoing outpatient cancer care and categorized 31.0% at risk for malnutrition [30]. We could confirm the high prevalence with 35.7% in our patients who are 65 or older. Therefore, more attention should be paid to elderly patients who are at a high risk of malnutrition.

We could not detect any differences regarding gender and malnutrition. Alvaro Sanz et al. showed that significantly more males were at risk for malnutrition [27, 31]. However, that study included of a large proportion of patients with breast cancer, who do not present the same nutritional risk as patients with other types of tumors.

We found that patients with risk of, or manifest malnutrition, according to the MNA also showed lower values for the global health status. Likewise, those patients have significantly lower functionality scores and a higher symptom burden. Considering side effects of cancer therapies, we have found loss of appetite to be the most frequent symptom associated with a decrease in food intake. This was consistent with results to several previous studies [28, 32–36]. Other symptoms often reported as reasons for diminished food intake were altered taste sensation and dry mouth [37], these are frequent side effects of several cancer therapies and so far, no truly effective supportive treatment exists. Further symptoms that seemed to increase the likelihood of reduced food intake are nausea and flatulence/diarrhea which likewise can be induced by radiation therapy or cancer drugs [38, 39]. In contrast, frequent nausea [40] is not associated with a higher risk for malnutrition but associated with a reduced QoL which is in accordance with the findings of Najafi et al. [41]. Interestingly, diarrhea did not show a significant association with malnutrition risk, while constipation was a symptom highly related for the risk of malnutrition. Still a study with patients surviving 2 years after gastric cancer resection showed an association between body weight loss and diarrhea [42].

Several short- and long-term health impacts are associated with malnutrition. Complications such as greater likelihood of length of reduced QoL [11], hospital stay [31, 43, 44] and costs [45] and other complications like postoperative complications [46], decreased response to treatment [47] and lower 90-day survival rate [44] and increased mortality [3] are connected to malnutrition.

Our data show that changes in nutrition habits are associated with loss of weight as well as different side effects such as alteration of taste or loss of appetite. This might be due to patients trying to react to nutrition problems [48]. It may also help physicians to identify patients at risk for malnutrition by routinely assessing side effects of cancer therapy [49]. On the other hand, our data might also point to an increased risk of malnutrition and side effects of cancer treatment resulting from nutritional changes, a hypothesis which should be assessed in further studies.

Several working groups were able to show that counseling by a trained dietitian is a cost effective method to reduce malnutrition [50–53]. Kufeldt et al. showed that individual nutritional support teams should treat patients identified as at risk for malnutrition. Patients should be identified and treated as early as possible which includes outpatient arrangements prior to (elective) treatment [54]. Tanaka et al. showed that nutritional counseling by a dietitian including information and education, combined with oral supplements and appropriate antiemetic treatment can prevent weight loss during chemotherapy [55]. Patients should receive individual nutrition advice adapted to their current situation, diagnosis, therapy strategies, prognosis, as well as sociocultural habits. In total, our data showed that less than a fifth of the patients got a nutritional counseling. Considering that nearly a half was at risk of malnutrition, this is a disturbing result pointing to an important deficit in the health care system. Maschke et al. reported that many cancer patients still lack the access to high quality nutrition therapy [15]. In contrast, patients are highly interested in the topic and more than half of our patients wanted to focus on nutrition. Patients with symptoms associated with eating are highly motivated which most probably would increase the impact of (early) nutritional counseling. In fact, our results indicate that systematic screening and early integration of nutritional management in clinical practice is indispensable for cancer patients independently of tumors entity. Furthermore, it may be valuable for all demographic subgroups. According to our findings, the medical care of malnutrition should include management of symptoms and side effects with the goal to stabilize and improve patients’ nutritional condition. This includes nutritional counseling and provision of proper information regarding questions concerning eating habits. This will not only help to reduce the risk of malnutrition, but at the same time may influence quality of life.

This study confirmed the high prevalence of malnutrition in cancer patients and its negative impact on quality of life. Furthermore, certain nutrition impact symptoms may indicate patients at risk even when a valid screening tool is not utilized. Despite the increasing data on malnutrition and its consequences on quality of life and prognosis [56] there still is a high prevalence of malnutrition and most patients do not get timely or sufficient support. Efforts to integrate nutritional screening and assessment into routine clinical practice for all cancer patients must be combined with a proper dietary consulting. Moreover, early information on nutrition should be offered independently of the current nutritional condition and patients should be encouraged to get involved in their nutritional matters and to closely cooperate with physicians and dietitians.

## Data Availability

Data may be obtained from the corresponding author.
